# Involving young people with lived experience in advancing mental health science: an exploratory qualitative study from Pakistan and India

**DOI:** 10.1186/s12888-025-07062-1

**Published:** 2025-07-01

**Authors:** Syed Usman Hamdani, Zill-e- Huma, Bhismadev Chakrabarti, Syeda Wajeeha Zafar, Ayella Gillani, Vaishali Bagrodia, Amy Finlay Jones

**Affiliations:** 1 Global Institute of Human Development, Shifa Tameer-e-Millat University, Pitras Bukhari Rd, H 8/4 H-8, Islamabad, Pakistan; 2 Department of Primary Care and Mental Health, University of Liverpool, Liverpool, UK; 3 School of Psychology & Clinical Language Sciences, University of Reading, Reading, UK; 4 India Autism Centre, Kolkata, India; 5 Department of Psychology, Ashoka University, Sonipat, India; 6 Telethon Kids Institute, Perth, Australia

**Keywords:** Young people, Lived experiences, Depression, Anxiety, Active Ingredients, LMICs

## Abstract

**Background:**

Meaningful involvement of young People with Lived Experience (PWLE) in co-designing youth mental health interventions has been much emphasized globally. However, there is a scarcity of evidence on involving PWLE of mental health problems in designing, implementing and evaluating mental health interventions, especially in Low- and Middle-Income Countries (LMICs). The aim of the current study was to understand the perspectives of young PWLE from two South Asian countries, Pakistan and India, regarding “Active Ingredients” (AIs) for youth mental health (i.e., components or processes of mental health intervention(s) that make a difference to mental health outcomes), as part of the Wellcome Trust AI Commission.

**Methods:**

For this exploratory qualitative study, we conducted 30 qualitative interviews via Zoom with young PWLE from Pakistan (*n* = 19, 14 females and 5 males) and India (*n* = 11, 8 females and 3 males) to explore their views about different AIs for youth anxiety and depression in South Asia. The qualitative data was analysed using a thematic analysis approach that moved through the phases of familiarization, generation of codes, searching, identification and review of themes and selection of illustrative quotes.

**Results:**

The results show that family and religion are integral to promoting positive youth mental health in the South Asian context. The AIs perceived to be most relevant for Pakistani and Indian young people were (i) improving social relationships; (ii) managing emotions; and (iii) relaxation techniques. Participants highlighted the need to explore the role of family support, personal space, spirituality/religion, schools, mental health literacy and stigma as potential AIs of mental health for young people in South Asia. The need for ease of access to mental health support and minimizing barriers to engagement with mental health services were highlighted as important contextual factors. Our findings highlight the need for culturally responsive youth mental health strategies that incorporate their preferred intervention components and address key challenges including stigma faced by South Asian youth.

**Conclusions:**

The current study highlights specific intervention components and contextual considerations that are important to Indian and Pakistani young PWLE when designing and delivering mental health interventions. Our findings underscore the need to work with young PWLE and consider their context, culture, and resources when developing or evaluating mental health interventions. Given our sample likely represents a relatively advantaged group, future studies can use targeted sampling strategies to capture perspectives of young people from lower socio-economic strata.

## Background

Emotional problems such as depression and anxiety are common and account for 45% of the global burden of the disease among young people, with a global economic cost of over $1 trillion per year [1]. Emotional problems in young people are predictive of mental health difficulties in adulthood [2], and are associated with significant impairment in academic performance and social problems and increased risk of suicide among youth [3]. There is a growing body of evidence showing effectiveness of psychosocial interventions and programs to improve mental health outcomes in young people; however, the science of *how*, *why* and *for whom* these psychosocial interventions work (i.e., which components of them make a difference, why these components are meaningful, and who they are meaningful for) is not well understood. Such information is important in designing and tailoring psychosocial interventions to particular target groups and conditions, optimizing youth engagement, and ensuring that scarce resource and efforts are efficiently used.

To address these gaps and identify the next generation of treatments and personalized intervention approaches, the Wellcome Trust launched an Active Ingredients Initiative in 2020. Active Ingredients (AIs) are conceptualised as theoretically driven components, targets, or mechanisms of youth mental health interventions considered the *“best bets”* to prevent, treat and manage anxiety and depression in youth, aged 14–24 years old, globally. In its first commission, the Wellcome Trust worked with the mental health science community to synthesise the evidence on 27 different AIs that were broadly categorized into six groups, including (i) behaviours and activities; (ii) beliefs and knowledge; (iii) brain/body functions; (iv) cognitive and attentional skills; (v) human connections; and (vi) socioeconomic factors [4].

While youth engagement strategies varied across the projects commissioned as part of the first round, involving young people in answering the research questions and interpreting the findings was a cornerstone of the AI approach. A key insight from the first AI Commission was that consulting young people with mental health problems is recommended to ensure youth mental health initiatives are co-designed with their involvement. Further, it was acknowledged that no single AI works alone – rather, there are bi- or multi-directional relationships between different AIs, which often share a high degree of conceptual overlap despite being supported by relatively distinct bodies of research. Importantly, the first round identified a dearth of evidence on the effectiveness of ingredients in low- and middle-income countries (LMICs), and the majority of stakeholders engaged in the evidence synthesis process were from high-income countries. This limits the generalizability of the insights gathered about which AIs are most important for achieving equitable impact on a global scale. This research was conducted as part of the Wellcome Trust-commissioned realist review of 27 AI reports from the first commission and sought insights from the young PWLE across six countries, including Australia, the UK, South Africa, Kenya, India, and Pakistan. This paper describes qualitative insights about 27 AIs from young PWLE with anxiety and/or depression in India and Pakistan.

South Asia, home to 613 million young people under the age of 18, accounts for more than a quarter of the world’s children [5]. Mental health problems (stress, anxiety and depression) are on rise among young people in South Asia [6]. Pakistan and India are South Asian neighbour countries. The combined population of two countries is about 1.5 billion people, of whom about half are under the age of 18 and three quarters live in rural areas. Literacy rates range between 55–60%, with lower rates among women. The two major religious traditions are Hinduism (80.5% in India and 1.16% in Pakistan [7]) and Islam (95% in Pakistan and 14.2% in India).^1^About 23.3% youth in India and 25% in Pakistan suffer from mental health problems respectively [8–10]. There is a rise in communicable diseases as well as in non-communicable diseases, giving the healthcare system a double burden. Despite this growing burden, the healthcare systems in these countries are already overstretched, facing a double burden of rising communicable and non-communicable diseases. At the same time, public spending on health in both countries is very low, with healthcare expenditures in 2018 amounting to just over 3% of Gross Domestic Product (GDP) in both Pakistan and India [11]. Less than 1% of the health budget is allocated to mental health care in Pakistan [12] and just over 1% in India [13]. The vast majority of the funds are spent in the area of physical health, and very little is allocated to mental health care. Outside a few major urban centres, there are no services of any description and very few mental health specialists are available to address young people’s anxiety and depression. Thus, the ‘treatment gap’ for anxiety and depression in the region is nearly 90% [14].

Understanding youth perspectives on mental health is a relatively nascent line of enquiry in South Asia. A recent systematic review of studies of Pakistani community perspectives on mental health found only two studies that focused specifically on youth living in Pakistan [15]. One of these focused on the perspectives of young people in an indigenous ethnic and religious minority (Kalasha) and identified distinct cultural protective factors for mental health, perspectives on causes of mental illness that included supernatural and spiritual factors, and preferences for treatment that included Shamanic practices, the use of amulets, and sharing and problem solving with the community [16]. The other found that among youth with a psychiatric diagnosis in Pakistan, participants identified their emotional state, family problems, and bad luck as playing a causal role in their mental health difficulties. A qualitative study by Parikh et al. [17] examined context-specific stressors among school-going adolescents in India, highlighting the role of proximal social environments (home, school, peers, and neighbourhood) in shaping stress experiences. Academic pressure, difficulties in romantic relationships, parental and peer influences, and exposure to violence were identified as key stressors. Gender-specific concerns such as conformity to societal roles and the risk of harassment were particularly specific stressors for girls. In India, a study with a stakeholder group of individuals working with youth found that stigma and limited mental health awareness were key barriers to young people accessing support [18]. Additionally, [19] identified key stressors for Indian adolescents, including family conflicts, academic pressures, peer relationships, and social position. Adolescents reported coping through rationalization, acceptance, distraction, spirituality, and self-comforting strategies. Another study from India [20] emphasized the importance of acknowledging young people’s contributions and ensuring contextual relevance when designing digital interventions in LMICs. Other studies have found that South Asian youth living in developed countries have poor awareness of available mental health services and options for seeking support for mental health concerns [21].

Building on this work, the aim of this study was to conduct qualitative inquiry with young PWLE in India and Pakistan, to understand their perspectives on the different AIs identified in the first commission, how they describe and prioritise them, and whether any important AIs were missing from the initial list. Participants were not only encouraged to offer insights grounded in their personal experiences and narratives related to mental health, but also to contribute perspectives derived from their engagements with such matters. Our specific research questions were as follows:

1. How do young people from Pakistan and India express stress, depression, and anxiety and what do they see as the key contributing factors in these experiences?
2. What are the views of young PWLE about addressing youth mental health problems in Pakistan and India?
3. From the perspective of young PWLE:
  - Which AIs identified in the first commission are considered most important for improving youth mental health in India and Pakistan and why?
  - Which additional AIs should be investigated and why?
  - What are the key contextual considerations (regional, religious, and cultural) for development and implementation of youth mental health services/programs in India and Pakistan?

## Methods

### Study design

It was a pragmatic exploratory qualitative study conducted by multidisciplinary experts with diverse backgrounds and expertise. AFJ (clinical psychologist and clinical academic) served as the Principal Investigator (PI) and provided overall guidance and leadership throughout the study. The consultation in Pakistan was led by UH (psychiatrist and global mental health expert) and ZeH (clinical psychologist and global mental health researcher). The team is leading the implementation of the ongoing President of Pakistan’s Program to promote youth mental health through schools in Pakistan [8, 9]. In India, BC (neuroscientist and autism researcher) along with VB (psychology researcher) at the India Autism Center led the consultations. Our multidisciplinary team of experts informed all phases of the research, including study conception, design of qualitative interview guides, analysis and interpretation of the qualitative data, write-up of study findings, and manuscript, ensuring a holistic understanding of youth mental health across disciplines, cultures, and countries. We conducted in-depth, semi-structured interviews with young PWLE in Pakistan and India. The interview guide was co-designed with input from young PWLE, and interviews were facilitated by researchers in Pakistan and India.

### Study participants and settings

Eligible participants were young people aged 14–24 years, who self-identified as having lived experience of stress, anxiety, and depression after responding to an invitation, sent via email for participation in the study. As the current study was a pragmatic qualitative study, no strict inclusion and exclusion criteria were used; therefore, the included sample might not be a complete representation of the socio-economic and health demographics of both countries. Since the study was conducted during COVID-19 pandemic, only those adolescents who had access to emails and were/had experience of seeking mental health care from psychiatrists and psychologists were included in the study. Therefore, the study sample in India and Pakistan were drawn from major cities and presented middle-to high-income strata. The Indian sample was geographically diverse, and participants were recruited from 4 big cities of India (New Delhi, Mumbai, Kolkata and Bengaluru). Notably though, all participants belonged to middle-high socioeconomic status (defined by a combination of education, income, and occupation). All participants were educated from or were studying at private schools in India in one of the aforementioned metropolitan cities. Their family income was above Rs 600,000 INR per annum at the minimum and their parents were either businessmen or professionals such as doctors, engineers, or lawyers.

Participants in Pakistan were recruited from the twin cities of Rawalpindi and Islamabad (the capital city of Pakistan). They were high school or undergraduate students between the ages of 16 and 22 and belonged to working-class professional families (their parents were either doctors or academics in educational settings).

As part of the present work, consultations were conducted with young PWLE in multiple countries, including Australia, the UK, Kenya, South Africa, India, and Pakistan. However, it is important to note that ethical approval was obtained solely for conducting and publishing the insights derived from consultations in India and Pakistan. The ethics approval of consulting young PWLE in India and Pakistan was obtained from the Institutional Review Board of the Global Institute of Human Development (GIHD) Pakistan and the Ethics Committee of Ashoka University, India as well as from the ethics committee of the Telethon Kids Institute, Australia. Informed consent was obtained from the participants prior to any data collection.

### Recruitment procedure

Young PWLE of mental health problems were recruited using a purposive sampling technique. In Pakistan, participants were recruited through existing on-going President of Pakistan’s Program in Rawalpindi district of Pakistan [8] and through networks of psychiatrists and clinical psychologists working in both these cities. Potential participants were invited by the research team via email. In India, the potential participants were identified through networks of psychiatrists and clinical psychologists working in these cities. The research team sent them a copy of the electronic Participant Information Sheet. Before participation in the study, participants were invited to discuss the study with a member of the research team via Zoom if they wished to do so. All those who expressed their willingness to be part of the study were asked to sign the electronic consent form to indicate their consent and send the signed copy back to the research team. In the case of minors, an electronic consent form was shared with parents and/or guardians. Once parental consent and/or assent from the minors was obtained, the research team contacted the participant to arrange an interview as per the availability of the study participants. Only those participants who voluntarily agreed to take part and who signed the electronic consent form were interviewed. In Pakistan, interviews were conducted and transcribed in Urdu language (national language of Pakistan). For each consultation, participants were compensated with an incentive of Rs. 400 (PKR) for their time. In India, interviews were conducted and transcribed in English language. For each consultation, participants were compensated with an Amazon gift voucher worth Rs. 1000 (INR) for their time.

### Data collection

Semi-structured interview guides were prepared based on the research questions and pilot tested for feasibility and appropriateness in both countries with young PWLE (*n* = 5), before qualitative interviews. Our consultations were guided by the Youth Engagement Framework to ensure meaningful engagement of young people in all phases of the research. The framework aims to address barriers to engaging them, including a lack of mental health literacy, stigma associated with mental health difficulties, appropriate engagement methods, and safety concerns. Following these principles, we co-developed the interview guide and interview process with our youth advisors by conducting informal and formal meetings to reflect and share feedback on i) the meaning and phrasing of the questions used in the topic guide according to their context; ii) the stigma attached to the language used and how this can be mitigated; iii) the potential risk of emotional harm (re-traumatization, stigmatization) to young people from engaging in the consultation process, and appropriate culturally specific methods of engaging young people in the consultation; and iv) how best youth voices can be respected, accurately represented, and elevated. Furthermore, to ensure participant well-being during the interviews, several safeguards were implemented. Interviewers were trained to recognize signs of emotional distress and respond sensitively, including offering breaks or stopping the interview if needed. Participants were informed that they could pause or withdraw from the study at any point without any consequences. Additionally, a list of locally available mental health resources and support services was provided to participants who expressed distress or requested further assistance. These measures were designed to minimize potential harm and ensure a safe and supportive environment for participants to share their experiences. The pilot interviews were conducted using the draft topic guide prepared for the main interviews, and the feedback was reviewed in a post-interview reflective session among the study investigators. This process helped us ensure that each phase of the research was co-designed with young people, following the youth engagement principles and methods outlined in the youth engagement framework.

As the current study was conducted amid the COVID-19 pandemic, semi-structured qualitative interviews were conducted online via Zoom. These consultations were facilitated with a consultation website, containing a short video explaining the Active Ingredient (AI) approach and a brief description along with illustrative images of each active ingredient. The website was designed to facilitate participant engagement and ensure that participants were well-informed about the study's objectives.

All interviews were audio recorded. Each recorded interview was transcribed verbatim by the same team member who conducted the qualitative interviews. The transcriptions were later translated into English (only for those interviews which were conducted in Urdu) and reviewed to maintain the accuracy of verbatim. Data was collected until the saturation point was achieved at each site. The criteria for saturation included the absence of new information emerging from our data analysis. After conducting qualitative analysis, young PWLE who had given their consent for further contact were invited to participate in a reflective session with the research team. The purpose of this session was to discuss and reflect upon how their insights were analyzed and subsequently contributed to the consolidation of findings, to complete the feedback loop. Precautions against loss of or breach of collected study data included making sure that no study data forms or files ever contain identifying information and that linkage files were protected separately from study data (for electronic files, including password protection and encryption, and for physical files, storage in a locked cabinet with restricted access). In accordance with the guidelines of relevant IRBs for research involving minors, all study data will be retained until seven years after the child participant reaches the age of 18.

### Data analysis

Given the authors’ involvement in data collection, interview transcription, and leading the analysis, it was imperative to address any potential biases that may have arisen from their involvement. The authors were also cognizant of their pre-existing theoretical notions about young people's mental health problems and their experiences in their respective countries. In response, the authors prioritized reflexivity [22] during discussions, aiming to refine interview techniques and critically examine researcher interpretations of the findings. This involved meticulously reviewing the initial interviews prior to conducting subsequent ones and a thorough evaluation of interview technique.

The qualitative data was analysed using six-step thematic analysis process, outlined by Braun and Clarke (2006). This involves (1) familiarization with data; (2) generating initial codes; (3) searching for themes; (4) reviewing themes; (5) defining and naming themes and identifying relevant illustrative quotes; and (6) summarizing and writing the results. Transcripts were reviewed several times to achieve data familiarization. To integrate thematic analysis with the AI narrative, we employed both a deductive and an inductive approach. A preliminary coding structure was developed using a deductive method (utilizing an existing framework of active ingredients to guide the identification and analysis of themes related to the most and least preferred AIs), with an iterative (data-driven) approach used to further refine and validate the structure. Concurrently, an inductive approach was employed to identify and analysze themes that were related to views about addressing youth mental health in both countries, ensuring a comprehensive and flexible coding structure. Interview transcripts were double-coded to enhance methodological rigor. The themes were compared and discussed. Any discrepancies in coding were discussed and resolved through consensus, ensuring that interpretations were aligned with the data. The final coding framework was developed based on the frequency and qualitative relationships of the identified codes after discussion and consensus. Throughout the analysis phase, the authors actively explored their own interpretations of the data content in relation to the participants' actual verbatim. The authors frequently referred back to the original transcripts to verify that the analysis results remained faithful to the raw data. This collaborative process served as an additional check to identify and rectify any potential biases in the formulation of findings, further contributing to the study's methodological integrity and minimizing the risk of research bias while enhancing the study's trustworthiness.

### Safety procedures

Prior to engaging young people in each country, we liaised with local researchers, advocates, and service providers to identify local norms for accessible and culturally secure engagement with youth. These guidelines were integrated into the Youth Engagement Framework to help us to address some of the barriers of engaging youth with lived experience of mental health problems including lack of mental health literacy; stigma associated with mental health difficulties. Our strategies included consulting young PWLE about the appropriateness of engagement methods prior to conducting the research; ensuring a safeguarding procedure was in place for each country, compensating participants’ time; ensuring support pathways were identified; engaging cultural navigators where required, and ensuring that the engagement principles are communicated and adhered to by the interviewers.

## Results

A total of 30 qualitative interviews in Pakistan (*n* = 19) and India (*n* = 11) were conducted between 28th Dec 2020 to 9th Jan 2021 and 12th November to 28th December 2020 respectively.

The mean age of the study participants was 20.6 (± 2.99) years. About 70% (24/30) of the participants were young females. At the time of the study, only 27% participants (4/19 in Pakistan and 4/11 in India) were seeking psychiatric care services for their emotional problems. The characteristics of the participants are presented in Table 1. The average duration of the interviews was between 55 (± 7) minutes in both sites.

**Table 1:** Characteristics of study participants (*N* = 30)

Variables	*Total* *f (%)*	*Pakistan* *f (%)*	*India* *f (%)*
**Age in years (M[SD])**	20.6 [2.99]	21.26 (3.05)	19.6[2.87]
**Gender**			
Female	22 (70%)	14 (74%)	8(73%)
Male	8 (30%)	5 (26%)	3 (27%)
**Education**			
Matriculation (grade 10)	2 (7%)	-	2 (18%)
Intermediate (grade 11–12)	3 (10%)	1 (4%)	2 (18%)
Undergraduate (grade 13–14)	25 (83%)	24 (96%)	7 (64%)
**Religion**			
Muslims	19 (63%)	19 (100%)	-
Hindu	11 (37%)	-	11 (100%)
**Currently seeking outpatient psychiatric care services**			
Yes	8 (27%)	4 (21%)	4 (36%)
No	22 (73%)	15 (79%)	7 (64%)

The themes generated from the semi-structured interviews are as follows:

### Perceived sources of stress, anxiety and depression in young people in India and Pakistan

Qualitative consultations with young PWLE identified several social and family factors as perceived sources of stress, anxiety, and depression in young people. At the social level, unrealistically high expectations of society from young people; financial constraints and lack of employment opportunities for youth in their countries were identified by participants as contributing to mental health concerns. Further, a significant contributing factor was the impact of the COVID-19 pandemic, which, in addition to creating psychological stress, was also seen contribute to mental health problems by limiting contact with schools and social supports. “*I feel like these problems are at their peak right now because it is really difficult for some young people to get out of that (situation). They don't have like an escape; when school/colleges were open, youth went there and found an escape that way but now I feel like they have to face their problems (at home) because they don’t have an escape from it”* (Female, 22; Pakistan).

Across both countries, participants noted that stigma is a barrier to mental health care, preventing adolescents from talking about their struggles or seeking appropriate help. *“I think the barriers we face are, first of all, the stigma surrounding mental health. Even though I think it's starting to change a little bit. I don't think that change is coming fast enough or intensely enough”* (Female, 21, India). Stigma and lack of awareness about youth mental health problems were described as a self-perpetuating cycle, driving underreporting of concerns and undermining service access, which in turn prevented these problems from being recognised and addressed on a broader scale. *“In Pakistan, the issue of mental health among youth cannot be quantified accurately because many young people cannot access the mental health doctors and facilities to address their mental health problems. There are many people who suffer from mental illness, but they do not have services, or they don’t access the mental health doctors”* (Male, 21; Pakistan).

On the contrary, participants reported that social media dialogue about mental health problems is helping to raise awareness, but the information was perceived to be ‘*unreliable*’ and there is misconception about manifestation of mental health illnesses due to which sometimes people overrate normal emotional reactions as mental health problems. *"I guess now people have awareness about it through social media and other platforms; we see posts about it, and mostly people know about it. There is now so much information out there that people confuse their emotions with mental illness. If anyone feels stressed during a stressful period, then they think they are having an anxiety disorder. Because so much awareness is spreading all over the world, but this information is not from a proper authentic resource or from a therapist, people are confusing their periods of stress or sadness with mental illness. They are not able to see a line between normal emotions and mental illness" (Female, 15; Pakistan).*

At the family level, participants identified pressure from family members and poor family relationships as important contributing factors to mental health concerns. Specifically, unrealistic academic demands were highlighted as a risk factor for causing stress in young people. Poor communication among family members further contribute to escalate the stress among young people. *“…families should not pressurize children about their grades as students know the impact of good and bad grades on their future. Parents should let their children study what they want to study. Most families force children to study science, however, most of the children have an interest in the arts so they feel forced to study sciences… So, one should not pressurize them in that way”* (Female, 16; Pakistan). Stigma was also described as negatively affecting young people’s capacity to disclose mental health concerns within their family. *"In our community, we don't really talk about mental health, if you talk about someone’s mental health, people would be like, oh, this guy might be too weak as a person, and his condition might not be considered a real issue. People just think that it (mental illness) is just something you make up in your mind. Like, people might be uncomfortable talking about (mental health) with other people, including their own parents" (Female, 24; India).* Many participants perceived “*suppression of emotions*” as a cause of stress in young people.

### Views about addressing youth mental health problems in Pakistan and India

Participant’ perspectives on how to effectively address mental health problems in young people also reflected the need for multiple levels of intervention. At the individual level, having positive characteristics of self-awareness such as being able to rely on oneself to manage one’s own life (‘*self-reliance*’) and being aware of ones’ own actions were considered important for self-improvement (‘*agency*’); to build a positive self-image and to positively promote mental health in young people. *"You have to have self-discipline in your life. You need to organize what you want in your life, what you want to achieve, how you want to feel, what you want to have in your relationships, have self-discipline, and know what you want……..And when I say self-discipline, I mean organize your life; you know what you need to achieve, make plans for it, and go by your plans like making future plans. I think you can achieve whatever it is, no matter what goal it is. No matter if it’s a health goal or a social goal, whatever it is, you need to break it down, make a mind map, go by it, and execute the plan that will prevent so many problems in your life" (Female, 24; Pakistan).* In both countries, increased physical activity in young people was also highlighted as an individual protective factor.

Both in India and Pakistan, the role of immediate family support was emphasized. *"First of all, from very childhood, the child’s upbringing should be in such a manner that the child should be comfortable within the family and parents to discuss the problems. When young people keep problems to themselves, it results in mental health issues”* (Male, 21; Pakistan). Having healthy relationships with parents, specifically having an open and good communication among family members and less academic pressure from parents on youth, was identified as protective factor to promote positive mental health in young people. However, invalidation of young people’s feelings by immediate family members was highlighted as a risk factor for youth mental health illness. "*So, I really think that the first step is to just understand that it's okay to be mentally ill. And it's the same as being physically ill”* (Female, 21; India). Having a strong support network consisting of family and friends has been identified as having a positive influence on young people’s wellbeing, as it provides an avenue for sharing problems and fosters a sense of belonging and support.

Young people reported that raising mental health awareness and addressing mental health stigma among immediate family is important as parents lack the knowledge on youth mental health problems and are not aware of how to help young people to deal with emotional challenges (that are often characterized as ‘*dramatic*’ ‘*unreal*’ and ‘*self-created*’ by the parents and the family) and this sometime lead to non-recognition of emotional problems in young people. In India, one participant highlighted lack of mental health awareness among older generations as a barrier to young people receiving adequate support.

Another barrier is that older generations such as families and teachers are not willing to understand and accept these problems. *“So, education on mental health literacy for not only the youth but also for the older generations can help the youth feel better” (*Female, 20; India).

Many young people reported having ‘*someone to talk to*’ and ‘*being able to talk about one’s own feeling*’ is the single most important factor to protect against developing distress in young people. Role of peers was frequently reported to be important in this regard. “I *guess the important thing is that there is someone who is always there for you to talk to, whether it's a family member, a friend, or a therapist or anyone else, so at least there is someone to talk to and say everything that is going on with you without being judged" (Female, 20; India).*

The role of social media was reported as “double-edged sword”; where on one hand social media usage was perceived to be helpful in raising mental health awareness among communities, its excessive use was identified as a potential risk factor that is negatively impacting young people’s self-worth and wellbeing by creating ‘*unrealistic expectations from life’*. Participants noted that excessive social media use contributed to unrealistic life expectations and negative self-worth, highlighting its potential to both support and harm youth mental health. These findings align with prior research, emphasizing the need for digital literacy and mental health education to help young people critically evaluate the information they consume online. At the school level, helping young people to build their character and teaching them life skills were considered important to promote mental health in young people.

In both countries having an employment opportunity for them were also highlighted as protective factors. There was an expectation that it is the government responsibility to “*organize extra-curricular activities at school or community” levels* and “*offer employment opportunities*” to young people to promote positive mental health.

### How to address mental health problems in youth

All young people reported ‘active ingredients’ as relatable, valid, comprehensive, and helpful to prevent and treat youth anxiety and depression. There was an enthusiasm in young people to learn more about AIs initiative. Young people in India described Active Ingredients as anything (‘*small components*’) that can help young people to feel good, keep them mentally stable and promote positive mental health in young people.

#### Improving social connection and relationship

For most of the young people, increasing social connections and improving social relationships (i.e., strengthening healthy relationships with family members) was highlighted as the most important AI. According to young people, youth in Pakistan live in a collectivistic culture that values family cohesion, solidarity, cooperation and good social relations, therefore social cohesion can positively impact the mental health of young people. Also, due to increased social isolation and loneliness because of pandemic and COVID-19 pandemic, the need for more positive social interaction was highlighted as an integral component to promote positive wellbeing. One young person (24, Male) from Pakistan said: *“I think that social relationships will be very high on my list as social relationships are very important, especially from a Pakistani perspective where our society and our family units are more cohesive, there's a greater degree of co-dependence and that might seem like a hindrance, at times, but in times of crisis like if you can leverage your social networks, people will mobilize to help you if they know that you need help and if they understand the importance of what you're saying."*

#### Improved management of emotions

Participants highlighted that management of emotions was important given the socialisation of emotion in India and Pakistan. Many highlighted how cultural expectations discourage emotional expression, contributing to mental health challenges. A 16-year-old female participant from Pakistan noted that young people often struggle with emotional regulation because societal norms discourage open expression: "*Young people should learn skills to manage their emotions. They don’t know how to regulate their emotions and express them because, in our society, expressing emotions is considered wrong, which is also a cause of depression and anxiety. Our society portrays that strongmen or boys don’t cry, their feelings can’t be hurt, and these are all wrong things. People should understand that apart from being men or boys, they are humans, they have emotions, and they can express them. If someone shows their emotions, people degrade him so much that he starts to think that he did something wrong."*

Similarly, an 18-year-old female participant from India reflected on the lack of awareness around emotional well-being and its consequences:

*"We never talk about what our emotions are and how we feel. We don't know how to manage things (emotions). If I was taught how to cope with stress and that stress is normal, then I wouldn't have had panic attacks."*

#### Better stress response by relaxation

Many young people regarded relaxation techniques as simple, easy and effective (*“…[meditation] does relax you and …makes it like more, um, attentive to your …, not thoughts per say, but like your sensory feelings, Male 22, India*)” and highlighted to be taught in schools to promote positive wellbeing in young people.

"Relaxation techniques should also be implemented; these techniques should be done especially in schools, universities, and colleges. I think private schools are now focusing on these. They should take the initiative to focus on these more” (Female, 22; Pakistan).

Some Indian respondents mentioned better stress response by relaxation as an available resource which is an integral characteristic of an AI:

"I think an important characteristic of an active ingredient is its availability. If it's something like relaxation or sleep, if you're doing a job where you don't have time to relax or sleep, then what are you going to do? So that's important. I think it doesn't have to be something that you need to buy" (Female, 21; India).

#### Access to urban green spaces

Young people highlighted that, in South Asian culture, female adolescents have far less access to green spaces compared to male adolescents. Access to green spaces is a novel and important component that is underutilized currently; however, can be considered crucial for the prevention of mental health problems in adolescents.

"Along with these, green spaces and neighbourhood cohesion are very important for prevention because I have personally felt that the mind feels fresh while seeing the greenery or natural environment; we get a sense of recess or feel easy, even though working in a close room feels like a depressive environment to me, and I get a fresher feeling by seeing the sunlight of the day" (Male, 21; Pakistan).

"Although I really want to go to the park every evening and enjoy green spaces, you know that females cannot go outside alone because of security reasons. Parents are afraid of sending us alone without a brother" (Female, 21; Pakistan).

### Least likable AIs

Active Ingredients that aligned with the way that young people understand their mental health were more likely to be perceived as relevant (such as social relationships and improved management of emotions), while AIs that did not work for them in the past or they were not aware of were consistently rated as the least preferred (such as anti-depressants and cash transfers). Young people spoke about personal experience as the dominant factor in guiding their decisions about which Active Ingredients were likely to be most helpful for them. Personal experience was either their own experience of what had worked or not worked for them in the past or the experiences of close friends and family members who suffered from mental health problems.

### Missing components in the current AIs list

#### Role of family and parenting

Family-based and parenting components were described as an important missing AIs by young people in both countries. It was emphasised that parenting and family-focused programs are particularly important in the South Asian culture. Maintaining healthy boundaries and developing effective communication with parents and other family members was considered important to positively influence mental health of young people.

A 21-year-old female from Northern India highlighted that the importance of parenting and family interventions:

"The effects of parenting on youth are profound- especially in the context of India, because our lives are so intertwined with them. [Young people] need help figuring out how to create healthy relationships with [their families] or to learn how to create boundaries. [They] need to create boundaries, not just with their parents but with everyone, especially with Indians, because our culture is so friendly and interdependent, that we don’t know how to draw the line. This is really, really important learning how to say no, respecting your own capabilities and limits, and knowing where to stop” (Female, 21; India).

Multiple pathways were highlighted through which family and parenting interventions may influence mental health of young people, including reducing risk factors such as parent mental health problems and child maltreatment, increasing protective factors such as attachment and positive parenting skills, supporting implementation through increasing engagement in interventions, and increasing the strength of intervention effects.

"Improving parent–child relationship. I think those should definitely be added because parents are your very first interaction with the world. You grow up with them. And you should be able to share whatever you can with them, I think, especially in our culture; in a ‘Desi culture’, parents usually don’t involve their children and communicate with them. But I do believe that, after a certain period of time, you do come to an understanding (with them), but I feel understanding should be built earlier, and one should definitely be able to communicate with them (parents) openly in every matter" (Female, 22; Pakistan).

#### Respecting personal space/safe spaces

In both countries, young people mentioned the importance of feeling safe. They emphasized the need for both a physically safe environment as well as safe spaces to discuss difficult issues. While the former was described as partly reflected in the Neighbourhood Cohesion AI, safe spaces were seen as an important AI in their own right. The importance of safe spaces was reiterated by young people. *"I don’t live in a kind of neighbourhood where I can just go for a walk outside, so I think this issue is important for, especially, women’s mental health" (Female, 20; Pakistan).*

"When you're Indian and you're living at home with a lot of people (extended family). They are an important part of your day-to-day activities and your environment. A lot of your activities are influenced by your parents or extended family. I think Indian families are much more involved in their kids’ lives" (Female, 20; India).

In addition to physical safety, young people in our consultations described interpersonal safety as an essential precursor to willingness to disclose mental health difficulties and seek help. Safety was emphasized as a key characteristic of effective mental health services for young PWLE, especially in Pakistan where young people spoke about a mistrust of medical professionals and a preference for peer-delivered models of psychosocial support. Fluid personal boundaries in collectivist Asian countries was reported to be an important risk factor to mental illness among young people. Respecting young people’s personal space was considered essential to create safe space for them.

"Personal space is like the space you need to breathe and the space you need to exist. It is a major thing … because in our Pakistani society, we are like a collectivist culture, so everyone needs to know everything about you. Even if it's your parents, siblings, or friends, everyone makes you feel like you owe everyone an explanation. People need to realize that sometimes it is better to spare another person" (Female, 22; Pakistan).

#### Religion and spirituality

Religion and spirituality were highlighted as a potentially important Active Ingredients that is currently missing in the AIs list. It was recognised that religion could act as both a facilitator and barrier for improved mental health outcomes in young people. Over-reliance on religion as a source of mental health support was also highlighted to be due to stigma and fear of being judged in seeking professional mental health support in South Asian culture:

"I told my teacher I am on medication, and I am having a lot of problems. I was told it is (taking medications) a negative trap, that if you follow religious practices, everything will be fine; you don’t need services. When people, elders, and teachers give such a strong negative response and forbid you (from seeking professional mental health support), then it becomes a hindrance to overcome mental health problems" (Female, 15; Pakistan).

Given the pervasiveness of religion in the context of South Asian subcontinent, young people also felt it important to consider how religion and spirituality might be harnessed to promote youth mental health. *“I think religious factors could also be used as a positive influence. In our country, there are so many people who are uneducated and rely completely on religious education. So, considering this, you can use religion as a positive influencer, like in the case of suicide. One should not commit suicide, as it is haram (forbidden in Islam) and shouldn't be committed” (Female, 22; Pakistan).*

“I mean, there are definitely cultural barriers in-play, like mental health. I think historically what's happened is that religion has played an important role in mental health, and that's where you meditate and pray and regain your sanity. And that's where people feel like they can get a sense of groundedness and a sense of stability/security. But with the next generation and people becoming less religious, you know, religion is becoming less of an important factor in people's lives” (Female 23, India).

#### Mental health literacy and stigma reduction

Mental health literacy was another important active ingredient raised by many of the young people that is currently missing from the AIs list. Across both countries, agency was linked to both mental health literacy (i.e., knowing what mental health difficulties are and how to seek support) as well as willingness to be vulnerable.

"One thing that is not on the list, now that I think (is important) is ‘information’, just by being informed about what mental health is, you know when to reach out for help when things are not going well in your life. That I think is something that’s missing in our society generally" (Female, 20; India).

Young people also raised the issue of misinformation around mental health symptoms and the importance of being able to distinguish between healthy emotional responses and signs of mental illness:

"I guess now people have awareness about it through social media and other platforms; we see posts about it, and mostly people know about it. Now there is so much information out there that people confuse their emotions with mental illness" (Female, 15; Pakistan).

#### Role of school

Young people emphasised the importance of schools as a setting for the delivery of mental health supports across the spectrum of mental health promotion, prevention to clinical interventions. A need for easily accessible mental health services in schools and educational settings was raised as a priority by all young people.

"I honestly don't think that schools pay enough attention to children's mental health, at least mine (school). It's a great school; we have a school counsellor as well, but I think when it comes to anxiety, depression, or any such thing that a student might be facing, they just shut it out. and I think that our age group needs somebody to reach out to us, someone we can communicate with" (Female, 16; India).

In Pakistan young people particularly emphasized the importance of school-based approaches particularly in the context of family and academic pressures, and to raise awareness of promoting mental health awareness and stigma reduction among the families and school authorities.

### Cultural considerations of implementing Active Ingredients in South Asia

Across our interviews, young people frequently highlighted the importance of the family environment as a key contextual factor influencing youth mental health. Family pressures were perceived as one of the primary stressors for young people, and a lack of family awareness about mental health issues was described as invalidating and undermining mental health supports.

“I think there is a need to address it because of generation gap, communication gap is increasing. Parents are saying that if you do such things, you will become a better human, but for children, these things may not matter. So, ultimately, children feel that their parents don’t understand them” (Female, 23; Pakistan).

The role of family awareness and support was also linked to stigma around mental illness, which was identified as a major barrier to help-seeking and provision of mental health care. Many young people highlighted the issue of mental health stigma, particularly within their families and school environments as a key barrier in accessing mental health services. According to a young person from India “…*the biggest barrier (to seek mental health care) is the culture in which we've been brought up. Like, I would say that the culture was never supportive (to seek professional help for mental health problems). We were never told that it's okay to seek help or it's okay to attend counselling sessions or attend therapy sessions. So, there's a taboo, there's a huge taboo of like,"Oh, you're getting help,"and people are judging you on it” (Female, 18’ India).* Interventions to normalize mental health difficulties and reduce stigma were considered crucial for effective implementation of mental health interventions.

Demographic factors also play a unique role within the cultures we explored in this study. In our data, gender was identified to play as an important role both in the sorts of stressors young people are exposed to, and the types of solutions and services that are likely to be accessible to male and female young people in India and Pakistan:

"Boys can go out any time. [Girls] can’t go out at any time of the day or be with their friends and talk about things. For them, they have to stay at home, and they can go out with their parents or guardians. What helps me is when I am feeling anxious, I want to go out and get some fresh air or do some activity, but I have to be with Mama and wait for her to be free? If I were abroad, I could go on my own, but here I can’t go alone for a walk or get some fresh air because it’s not safe. So, this is a big problem that you can’t even go to the parks, etcetera" (Female, 15; Pakistan).

Additionally, according to young people in Pakistan sources of anxiety, depression and stress in younger adolescents (aged 14 to 18 years) are comparatively different from older adolescents (aged 19 to 24). Younger adolescents are more likely to experience stress, anxiety and depression due to academic pressure whereas, the older adolescents, who are in the period of transition from school to work, tend to experience distress due to problems related to finances and employment. Therefore, implementation of AIs will require careful consideration regarding age of young people.

## Discussion

When designing improvements to health services and developing novel treatment options for a given condition, understanding the perspectives of service users and those with lived experience of the condition is essential to ensure outputs are engaging, meaningful and feasible. While there is a marked need for better access to mental health services for South Asian youth, there is a dearth of research exploring what matters to those with lived experience in this population. By documenting the perspectives of young PWLE in India and Pakistan, this study provides important insights into youth perceptions of mental health and the factors they consider most meaningful when improving youth mental health in India and Pakistan.

The factors that young people highlighted as negatively impacting their mental health, such as closure of schools due to COVID-19 pandemic; poor family relationships; excessive use of social media and technology; and unrealistically high expectations of society from young people, align with those identified in some prior studies in South Asian and in global youth mental health more broadly [23, 24]. Several studies around the world have highlighted the adverse consequences of closure of schools due to COVID-19 pandemic [25]. According to an estimate, around 91% of the world's student population has negatively been impacted by closure of schools and colleges due to COVID-19 pandemic [23]. The findings of a systematic review show that reduction in social connections and physical activities due to closure of school during COVID-19 pandemic resulted in harmful effects on mental health of young people and contributed to exacerbated rates of psychological health problems among them during COVID-19 [24].

Another perceived source of distress for Indian and Pakistani young people reported by our study participants was poor family relationships. Family stressors including poor family relationship (lack of warmth and affection) and parental expectations to academically perform better have been categorized as a ‘principal source of stress’ for young people in LMICs in the literature [26, 27]. In particular arguments and conflict with family has been identified as a threat to youth wellbeing and can have determinantal effects on their mental health in later life [27].

Other factors cited as playing a key role in mental health problems in our study included unrealistically high social expectations placed on young people, academic pressures, and lack of employment opportunities for young people in their countries. These factors represent contemporary challenges with specific manifestations in different countries. The findings of a recent systematic review by Renwick et al., [27] on the conceptualization of positive mental health in young people in LMICs identified that for younger adolescents, academic stress (e.g., academic overload and exam pressure) is a consistent and considerable source of stress, whereas older adolescents are under great amount of pressure for securing a job after successfully completing their studies in LMICs [27]. Prior work has emphasized the role of spiritual and religious influences on the mental health of South Asian communities [16]; these themes were also identified as prominent factors in the findings from the current study.

While reflecting on the AIs’ list, participants reported that the most preferred AIs for young people from both sites are improving social connection and relationships, management of emotions, relaxation techniques and access to urban green spaces. This resonates well with the existing evidence. For example, Renwick et al., [27] identified that quality of social relationship, especially relationship with family served as an effective coping strategy to deal with stress among young people in LMICs [27]. Conversely, interpersonal difficulties contribute to loneliness among young people, which in turn can play a role in precipitating, exacerbating, or maintaining mental health concerns. Managing emotions was the other preferred AI for youth in both sites. It could probably be because in collectivistic cultures, young people are expected to adhere to family, social and cultural norms and emotional suppression is more common in these societies than in individualist cultures. The findings of recent schools based mental health trials show that the skills training program containing the components of both emotional regulation and relaxation techniques is found to be acceptable for Pakistani young people to address both their family and academic problems [26, 28] as well as for Indian adolescents [17]. Additionally, a relaxation-based school program also resulted in reducing symptoms of anxiety among adolescents in Sri Lanka [29].

AIs that were not congruent with personal experiences or understanding of what is likely to be helpful (such as antidepressants, gut microbiome, and cash transfer) were generally dismissed by young people in our study. According to the participants, the most important active ingredients of youth mental health for India and Pakistan, that are currently missing in the AIs list, were role of family and parenting, respecting personal space, religious and spiritual support, mental health literacy and stigma reduction and role of schools. Socio-cultural context plays an integral role in understanding and managing mental health problems [30]. The conceptualization of mental health and wellbeing in young people from LMICs is informed by their psychosocial context in which they pay greater attention to family, religion and schools in the development as well as management of mental health problems [17]. Non-judgmental and close family relationship and religious and spiritual support have been frequently reported to act as a protective factor to cope with the stress among young people living in LMICs [31]. In this context, seeing mental health literacy as a multilateral concept can enhance family, religious, and educational support networks, promoting the holistic well-being of youth from low- and middle-income countries [32, 33].

Our findings also highlight the significant influence of perceived gender role expectations on young people’s mental health experiences and help-seeking behaviors. In South Asian contexts, societal norms and expectations impose distinct stressors on young males and females—such as mobility restrictions and heightened safety concerns for young females, and financial pressures and societal expectations of resilience from young males. These gender experiences shape how mental health challenges are perceived, expressed, and addressed within families and communities. Given that the 70% of our sample consisted of young females, the narratives captured may reflect a stronger emphasis on stressors commonly perceived by females, potentially limiting the visibility of challenges faced by young males. Future research should explore these gender roles and expectations further to ensure a more comprehensive understanding of youth mental health challenges and needs from a gender lens.

Across participants, need for ease of access to mental health support and minimizing barriers to engagement were highlighted as important considerations. Low mental health literacy and mental health stigma were consistently cited as key barriers to mental health service access, a finding that aligns with prior studies [30, 34, 35]. In a recent systematic review of studies of mental health stigma among Indian youth found that 30% of youth had limited knowledge of mental health symptoms and determinants and stigmatizing attitudes about people with mental health problems [36]. As highlighted in the present study, stigma amongst youth is compounded by multi-generational stigma that can serve to undermine or invalidate the experiences of young people with mental health concerns. This finding echoes the ‘collective stigma’ identified in a recent Indian study – which is distinct from the individual level stigma often reported in studies in the global north [32, 33]. Together, this finding highlights the need for targeted stigma-reduction efforts with messaging aimed at multiple generations, given that the perspectives of parents and grandparents may be more influential in the lives of South Asian youth than for young people in other parts of the world.

In addition to addressing stigma and mental health literacy, participants in our study suggested that ideally, mental health supports should be delivered within the naturalistic settings for young people such as social media platforms and educational settings such as schools. School-based mental health services in India are considered acceptable, with a focus on relational engagement, problem-solving interventions, blended self-care approaches, and context-specific implementation strategies [17]. This aligns with recent calls to move mental health care out of psychiatric settings and into the community in Pakistan [8, 9, 37]. Participants emphasized the need for global accessibility, both in terms of implementation feasibility and in terms of cross-cultural relevance of concepts or activities. Importantly, it was noted that some approaches to mental health promotion perceived to be highly accessible in some countries (for example, physical activity), were not considered accessible in Pakistan, where neighbourhoods were reported to be unsafe and gym memberships were considered expensive and inaccessible. This calls into question the “universality” of some approaches to mental health promotion and underscores the importance of working with youth to determine which approaches to mental health support are most feasible and acceptable given their cultural, social, and environmental context. While both countries share cultural similarities, there are notable differences in how mental health is perceived and addressed. In Pakistan, religion and family play a central role in mental health support, whereas in India, there is a growing awareness of mental health issues, particularly among urban youth. These differences highlight the need for tailored interventions that reflect the unique cultural contexts of each country.

### Strengths and limitations

A significant aspect of our study was utilizing a youth engagement framework to ensure meaningful involvement of young people. By conducting reflective sessions post-interview, we effectively closed the feedback loop. This approach, combined with avoiding jargon, created a supportive space that ensures equitable involvement of young people with lived experience in mental health sciences [38]. The biggest strength of the study is its significant impact on shaping the mental health sciences agenda through the AI commission. Consultations with young people with lived experience of mental health problems highlighted important AIs that were missing from the initial list of 27 AIs. This feedback led to the establishment of a second AI commission, which incorporated the insights from these young individuals and prioritized areas such as family support, cultural connection, mental health literacy, spiritual and religious beliefs, school connectedness, and peer support for further research.

Use of prescribed list of AIs to frame discussion encouraged broad discussion among many factors that may influence youth mental health but may have also influenced the sorts of responses young people provided about what matters for mental health. High mental health stigma and low mental health literacy in the countries from where we recruited our study participants, might have led to sampling bias. Also, our data reached saturation, which was assessed by reviewing transcripts for the emergence of new themes. After 30 interviews, no new themes were identified, indicating that saturation had been achieved. However, it is important to note that the homogeneity of the sample in terms of education and socioeconomic status may have contributed to reaching saturation for this sample. Moreover, while we reached data saturation with our sample size, there are several socioeconomic factors that influence mental health literacy and service access that would have also influenced who participated in our study and therefore limits the generalizability of the findings. Our sample likely represents a relatively advantaged group given both their mental health literacy and their connection to mental health treatment networks. This is particularly relevant for the Indian sample which was drawn from the middle to high income strata in four major cities, due to the recruitment strategy. Future studies need to address this limitation by using targeted sampling strategies and supporting engagement of socioeconomically disadvantage youth to capture perspectives of youth from lower socio-economic strata.

## Conclusion

In light of the above findings, it can be surmised that the involvement of people with lived experience is critical to shaping mental health agenda. The Active Ingredients (AIs) approach to understand the aspects of interventions that make the difference, is acceptable to both Indian and Pakistani young people, however, categorization and implementation of AIs should match the particular context (cultural, social, religious, and economic) and preferences of young people to answer what works for whom under what conditions and why to prevent and treat anxiety and depression among young people living in low resource settings.

## Data Availability

Data are available from the corresponding author upon reasonable request.

## Abbreviations

AIs: Active Ingredients
GIHD: Global Institute of Human Development
INR: Indian Rupee
LMICs: Low- and Middle-Income Countries
M: Mean
PWLE: People with Lived Experiences
PI: Principal Investigator
PKR: Pakistani Rupee
SD: Standard Deviation
UK: United Kingdom

## Footnotes

1 Statista (2024). Retrieved on March 28, 2025 from https://www.statista.com/statistics/702004/share-of-muslims-2011/
